# Dr. Frank Morris Ridley

**Published:** 1895-05

**Authors:** 


### Editorial.

### DR. FRANK MORRIS RIDLEY.

It is with great pleasure that we are able this month to present an excellent likeness of Dr. Frank M. Ridley, of LaGrange, the newly-elected president of the Medical Association of Georgia. Dr. Ridley's election to this honorable place in the Association was very gratifying to his friends throughout the State, and the convention gave evidence of his great popularity by the practically unanimous vote of that body.

Dr. Ridley was born in LaGrange, Ga., January 1st, 1856, and received his academic instruction here, afterwards completing his education at the University of Georgia. He graduated in medicine at Tulane University, New Orleans, March, 1879. Immediately after receiving his degree of Doctor of Medicine, he located in the place of his birth, and has built up a large and lucrative practice. Dr. Ridley is a long term member of the Medical Examining Board of Georgia and is the secretary and treasurer of that body.

While our new president enjoys the reputation of being a splendid physician, he is also one of the most eloquent speakers in the State, and has more than once charmed his hearers by his flowing rhetoric and exquisite word painting. Dr. Ridley is a power of strength, politically, in his district, the fourth, and we predict that he will grace the halls of Congress before many years have passed away.

